# The effects of visual context on visual-vestibular mismatch revealed by electrodermal and postural response measures

**DOI:** 10.1186/s12984-022-01093-5

**Published:** 2022-10-20

**Authors:** Doaa S. Al-Sharif, Carole A. Tucker, Donna L. Coffman, Emily A. Keshner

**Affiliations:** 1 Department of Health and Rehabilitation Sciences, Temple University, 1301 Cecil B. Moore Avenue, Philadelphia, PA 19122 USA; 2 Department of Epidemiology and Biostatistics, Temple University, Philadelphia, PA 19140 USA; 3 Present Address: Department of Physical Therapy, University of Texas Medical Branch, Galveston, TX 77555 USA; 4 Present Address: Department of Psychology, University of South Carolina, Columbia, SC 29208 USA

**Keywords:** Vestibular migraine, Autonomic nervous system, Virtual reality, Virtual environments, Intervention, Visual dependence

## Abstract

**Background:**

No objective criteria exist for diagnosis and treatment of visual-vestibular mismatch (VVM).

**Objective:**

To determine whether measures of electrodermal activity (EDA) and trunk acceleration will identify VVM when exposed to visual-vestibular conflict.

**Methods:**

A modified VVM questionnaire identified the presence of VVM (+ VVM) in 13 of 23 young adults (34 ± 8 years) diagnosed with vestibular migraine. Rod and frame tests and outcome measures for dizziness and mobility were administered. Participants stood on foam while viewing two immersive virtual environments. Trunk acceleration in three planes and electrodermal activity (EDA) were assessed with wearable sensors. Linear mixed effect (LME) models were used to examine magnitude and smoothness of trunk acceleration and tonic and phasic EDA. Welch’s t-test and associations between measures were assessed with a Pearson Correlation Coefficient. Effect sizes of group mean differences were calculated.

**Results:**

Greater than 80% of all participants were visually dependent. Outcome measures were significantly poorer in the + VVM group: tonic EDA was lower (p < 0.001) and phasic EDA higher (p < 0.001). Postural accelerations varied across groups; LME models indicated a relationship between visual context, postural, and ANS responses in the + VVM group.

**Conclusions:**

Lower tonic EDA with + VVM suggests canal-otolith dysfunction. The positive association between vertical acceleration, tonic EDA, and visual dependence suggests that increased vertical segmental adjustments are used to compensate. Visual context of the spatial environment emerged as an important control variable when testing or treating VVM.

## Introduction

Individuals presenting with non-specific dizziness are often sensitive to conflict between visual and vestibular signals. This sensitivity has been named in the International Classification of Vestibular Disorders by the Bárány Society as visual-vestibular mismatch (VVM) [1]. VVM is defined by a cluster of symptoms, including false sensations of motion or tilting of the visual surround and visual distortions (i.e., blur), that are visually-induced and result from vestibular pathology or an unresolved conflict between visual and vestibular stimuli [1].

Dizziness due to VVM can be a challenge for clinicians because of the absence of an objective biometric measure that identifies criteria for diagnosis and treatment [2, 3]. A validated assessment of VVM does exist [4], but it relies on anecdotal reports and has not yet been tested for reliability as a tool to identify presence of, or objectively characterize VVM. The lack of measurable information about the clinical progression of this disorder affects the quality of rehabilitative care [5, 6]. Individuals may present with varying severity of dizziness, and the effectiveness of a treatment intervention may be dependent on its intensity [7]. Thus, effective rehabilitation and treatment methods for individuals with VVM have not yet been designed.

Our prior research revealed that a large proportion of individuals diagnosed with vestibular migraine also test positive for VVM (57%) and visual dependency (42%) [8]. A strong association between dizziness, visual dependence, and VVM implies that dysfunction in the autonomic nervous system (ANS) might be contributing to these orientation disorders [9]. Dizziness, nausea, and light headedness are autonomic signs that are elicited when vestibular and visual stimuli are in conflict [10]. Moreover, individuals with peripheral and central vestibular dysfunction have been shown to exhibit symptoms and signs of autonomic dysfunction [10–13]. Electrolytic or chemical lesions in the caudal region of the medial vestibular nucleus reduced vestibular-elicited activity in sympathetic nerves. These results implicate the vestibular system in regulation of ANS activities that maintain the stability of the human body's internal environment in response to changes in external conditions [11, 14, 15].

Sympathetic ANS responses can be assessed by using measures of electrodermal activity (EDA; [16–19]). Physiologic and subjective findings of a strong relationship between ANS and vestibular symptoms suggest that tonic and phasic EDA responses could serve as objective measures of VVM when in environments presenting mismatched or conflicting vestibular and visual signals. Thus, we postulated that EDA would provide an insight into the modulation of central sympathetic activities in individuals with VVM [20].

Of course, the symptoms observed with VVM could be the result of a disorder in the vestibular system itself. Individuals with unilateral and bilateral vestibular hypofunction tend to exhibit greater postural sway than healthy controls [21]. Intimate connections between the vestibular nuclei and cerebellum, cerebellum and frontal eye fields, and vestibular nuclei and parietal lobe likely contribute to the dizziness and disorientation evoked by VVM [22–25]. We postulated that associating the EDA with measures of postural sway would provide insight about how vestibular and ANS mechanisms interrelate in response to a conflict in visual and vestibular inputs.

Our previous [26] study revealed significant differences in the EDA and postural measures of young adults with vestibular migraine compared with healthy young adults. In this study, we further explore the changes in postural sway and EDA in individuals with vestibular migraine when exposed to visual and vestibular conflict [27–30]. We hypothesized that individuals with vestibular migraine who also exhibit symptoms of VVM will present with increased EDA and increased postural sway compared to individuals with vestibular migraine without VVM when exposed to an immersive virtual reality (VR) environment that produces visual-vestibular conflict [31]. In the attempt to formulate an approach for future interventions, we also explored whether the environmental context (i.e., amorphous moving textures vs. meaningful moving images) altered the magnitude of the EDA and postural responses.

## Methods

### Subjects

This study was approved by the Temple University Institutional Review Board (protocol #25913) and the Ministry of Health of the Kingdom of Saudi Arabia (protocol # H-05-FT-083). The convenience sample consisted of 23 young adults, 14 females and 9 males, with a previous diagnosis of vestibular migraine (average age 34.74 ± 8 years) who presented to the outpatient Otoneurology and Emergency Departments at Hafer Al-Batin Central hospital between the period of December 2020 and February 2021. Data from the healthy participants have been previously reported [26].

Those willing to participate provided informed consent. Of those, 13 participants with vestibular migraine tested positive for VVM (+ VVM) and 10 tested negative for VVM (−VVM) on the Visual-Vestibular Mismatch Questionnaire (Table 1) [32]. In a separate visit, vestibulonystagmography (bi-thermal caloric, positional nystagmus, smooth pursuit, random saccade, gaze stability, optokinetic nystagmus, and oculomotor testing) was performed on all participants who experienced migraine. Values of the abnormal caloric testing result were established by the clinical laboratory as a directional preponderance of 25% or greater.

**Table 1:** Demographic and clinical characteristics of participants with vestibular migraine (n = 23)

Variable	+ VVM [13]	−VVM [10]
Gender		
Female	10 (77%)	4 (40%)
Male	3 (23%)	6 (60%)
Age (years)		
Mean ± SD	34 ± 9	34 ± 8
BMI (kg/m^2^)^***^		
Mean ± SD	30 ± 8	26 ± 5
Handedness		
Right-handed	13 (100%)	9 (90%)
Left-handed	** − **	1 (10%)
Rapid assessment of physical activity		
Active	4 (31%)	1 (10%)
Under active	8 (61%)	8 (80%)
Sedentary	1 (8%)	1 (10%)
Activities of balance confidence^***^		
Mean ± SD	71 ± 22	95 ± 12
RFT (visual dependency) Mean ± SD (angle deviation)^**^	14 ± 4	10 ± 4
Dependent	11 (85%)	8 (80%)
Non-dependent	2 (15%)	2 (20%)
Visual vertigo analog scale^***^		
Mean ± SD	52 ± 13	5 ± 10
Dizziness handicap inventory^***^		
Mean ± SD	48 ± 24	9 ± 19
Vertigo symptoms scale-short for^***^		
Mean ± SD	14 ± 7	5 ± 6
Origin		
Vestibular	7 (54%)	1 (10%)
Autonomic	6 (46%)	8 (80%)
Both	**–**	1 (10%)

Positive visual-vestibular mismatch (+ VVM); Negative visual-vestibular mismatch (−VVM); standard deviation (SD); *p < 0.05; **p < 0.01; *** p < 0.001

### Procedures

Participants stood on the center of a standard AIREX 20" × 16.4" × 2" balance pad (Advanced Medical Technology Inc., Watertown, MA) with their arms at their sides and their feet about shoulder-width apart. Participants were asked to maintain an upright standing position with their eyes open while wearing a head mounted display (HMD) and watching a virtual visual scene for 3 min. Each exposure to the dynamic visual environment was followed by a rest period of at least one min until any emerging symptoms of dizziness, nausea, or any discomfort were verbally reported as resolved. During the rest period, participants were seated and the HMD removed.

#### Virtual reality environment

Participants were exposed to a three-dimensional complex visual environment generated by the software PosturoVR 0.8.3 (Virtualis, France) projected on the Oculus Rift HMD (Oculus Rift, CA). The field of view (FOV) of this device is more than 90 deg horizontal (110 deg on the diagonal). Vision of the real world is completely blocked, thereby providing a strong sense of immersion.

**Fig. 1:**
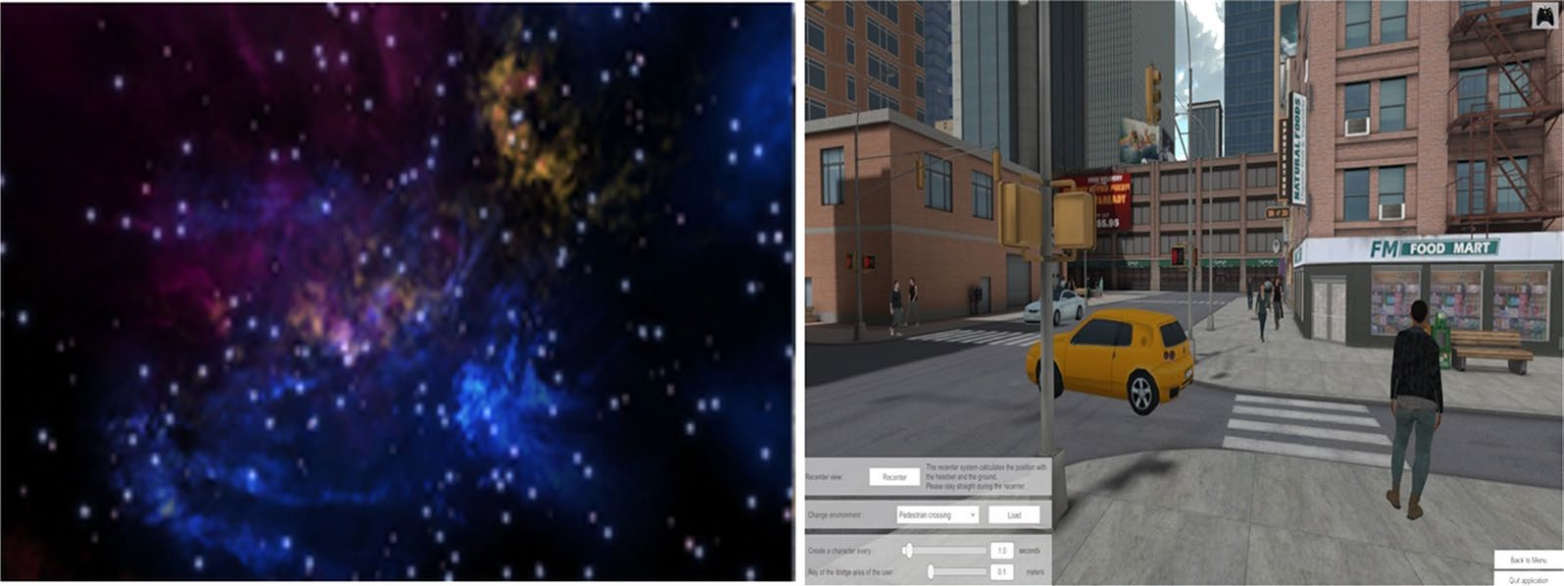
Images of the space (left) and street (right) virtual scenes

Two virtual environments (a space scene [SPACE] and a pedestrian crossing scene [STREET]) were randomly presented in one visit (Fig. 1). The space scene was a projection of star-like objects, at different sizes and distances from the participant, that rotated in the yaw axis with no cues indicating verticality. This image has been previously demonstrated to induce strong sensations of self-motion during quiet stance [33–35]. The direction of motion was in the direction identified by each participant as their dominant hand. The street crossing scene was constructed of three-dimensional, recognizable objects (i.e., buildings, sidewalks, traffic signals, cars, pedestrians) that moved in multiple directions at varied distances from the participant.

#### Electrodermal activity (EDA)

EDA is a measure of skin conductance and consists of a tonic component, also known as skin conductance level (SCL), which changes slowly over time (baseline) and reveals the active state of the sympathetic nervous system. A phasic component of the EDA, known as the skin conductance response (SCR), changes rapidly in response to external new, unexpected, and/or arousal-driven stimuli [17]. Sudden shifts of phasic activity above the tonic activity designate the SCR peaks.

Changes in EDA were recorded using the wireless Shimmer3 GSR + sensor unit (Shimmer-North America, Cambridge MA) that measures changes in skin conductivity produced by increases in the activity of sweat glands at a sampling rate of 128 Hz. The sensor was placed over the palmar surface of the medial metacarpal-phalanges of the third and fourth fingers of the non-dominant hand. Participants were instructed to close their eyes and relax until the investigator observed that activity detected and displayed by the Shimmer sensor unit remained close to a baseline.

#### Postural control

Trunk triaxial linear acceleration data were tracked with a Shimmer3 IMU wearable sensor with a sampling rate of 128 Hz placed over the L5 vertebral region.

#### Self-reported outcomes measures

The presence of VVM, dizziness, balance confidence, and the level of physical activity of each individual were evaluated at the beginning of the experiment using validated clinical tools. The Visual-Vestibular Mismatch Questionnaire (VVMQ) [32] presents situational questions to determine the presence of the cluster of symptoms that define VVM. The Visual Vertigo Analog Scale (VVAS) [36] ranks the intensity of dizziness in environments with dynamic visual input. The Dizziness Handicap Inventory (DHI) [37] quantifies the self-perceived impact of dizziness on activities of daily life. The Vertigo Symptoms Scale-Short Form (VSS-SF) [38] uses a five-point Likert scale to determine the frequency of symptoms. The Activities of Balance Confidence (ABC) scale [39] is a self-reported measure of balance confidence during various motor activities. The Rapid Assessment of Physical Activity [40] assesses the daily level of physical activity. Combined, these scales provide a general overview of whether dizziness and instability are affecting quality of life and daily functional activity.

The presence of visual dependency was confirmed with a Rod and Frame test (RFT) available on the PosturoVR 0.8.3 software (Virtualis, France) and projected on to the Oculus Rift [8]. At the beginning of each trial, the virtual rod was set randomly at a 45 deg angle to the left or right. The rod was then rotated manually (1 deg/button press) by the investigator toward a vertical position. Participants were instructed to raise their hand to signal when they perceived that the rod had achieved a vertical position. Throughout these trials, the contextual square frame was tilted 28 deg to the left. The same procedure was repeated four times and the measure of angular deviation from vertical averaged for later analysis.

### Data analyses

#### EDA measures

Raw EDA data was processed with MATLAB R2020b (The MathWorks, Inc., Natick, Massachusetts, USA) using the Ledalab-toolbox V3.4.9 (www.ledalab.de) through continuous decomposition analysis (CDA) to decompose the skin conductance data into its phasic (SCR) and tonic (SCL) components [41]. The CDA method can be applied to full-length data which provides a complete decomposition model of the original data. All mathematical models of CDA are based on a physiological rationale to avoid underestimation biases due to overlapping responses. However, the integrated skin conductance response (ISCR), defined as the area (time integral) of the phasic component within the response window, reflects the phasic EDA response to a given event or stimulus. It equals SCR multiplied by the size of the response window [Microsiemens ($\mathrm{\mu S})*\mathrm{seconds}(\mathrm{s}$)]. The detection threshold for significant peaks was set to 0.01 $\mathrm{\mu S}$ as recommended by the Society for Psychophysiological Research [18]. To prevent the common skewed distribution of electrodermal response measures, the standardized ISCR was computed as [41]:

$$ISCR=log(1+|ISCR|)\times sign(ISCR)$$

#### Postural acceleration measures

Trunk linear acceleration data was processed using MATLAB R2020b (The MathWorks, Inc., Natick, Massachusetts, USA) which provides a formula for calculating the Root Mean Square (RMS) and the Normalized Path Length (NPL). RMS and NPL were calculated for the antero-posterior (AP), medio-lateral (ML), and vertical (VERT) planes where a higher values indicate greater postural instability [42–45]. RMS is the mean power of the entire trial time and NPL is the sum of the absolute values of acceleration over time divided by the length of time that it takes to travel that distance, thus describing smoothness of the trunk motion. RMS and NPL were computed using the following formulae [45]:

$$RMS=\sqrt{\begin{array}{c}{\left(\begin{array}{c}\frac{\sum_{j=1}^{N-1}{p}_{j}}{N}\end{array}\right)}^{2}\end{array}} NPL=\frac{1}{t}\sum_{j=1}^{N-1}|{p}_{j+1}-{p}_{j}|$$

where $t$ is time duration, $N$ is the number of time samples, and ${p}_{j}$ is the acceleration data at time sample $j$. Data were low-pass filtered using a 4th order Butterworth filter with a cutoff frequency of 1.25 Hz. Each trial was plotted individually and inspected visually to ensure that the data were free from significant artifacts.

### Statistical analyses

EDA and six postural acceleration measures (RMS and NPL each in ML, AP, and VERT axes) were analyzed using R version 4.0.4 (R Foundation for Statistical Computing, Vienna, Austria). Correlations for continuous variables were computed with Pearson correlation coefficients with a two-tailed test. A Shapiro–Wilk test revealed the data were normally distributed.

Linear mixed-effect (LME) models were constructed to statistically assess the effects of the virtual visual environments (SPACE and STREET) across groups (+ VVM and −VVM) and time. Response variables included ISCR, NPL, and RMS with the subject as a random effect and a slope fit for each trial. LME models were fit using restricted maximum likelihood estimation [46]. After examining the full-effects model for EDA phasic, EDA tonic, RMS, and NPL responses in the AP, ML, and VERT planes, non-significant terms and interactions were removed. The final model for estimating the change in EDA phasic response included the interaction of group with time.

Specific differences between the virtual environments and groups were examined with a Wilcoxon signed rank test. Effect sizes were calculated using the following formula [47]:

$$\mathrm{r }=\mathrm{ Z}/\surd \mathrm{N}$$

where $r$ is the effect size, $Z$ is the Z statistic, $N$ is the sample size. Effect sizes were classified as follows: no effect (0.0 to < 0.1); small effect (0.1 to < 0.3); medium effect (0.3 to < 0.5); and large effect (≥ 0.5) [47]. For t-tests, the usual Cohen's d effect size measure was computed [47].

Data from self-reported outcome measures were analyzed using IBM SPSS Statistics v.23 (IBM Corporation, Armonk, N.Y., USA) and reported as mean ± standard deviation or as a percentage of participants. The significance level was set at *α* = 0.05 for all analyses. Bonferroni post-hoc adjustments were used to adjust for multiple comparisons. Differences in demographics and clinical outcome scores between the + VVM and −VVM groups were assessed using Welch’s t-test. Individuals were assigned positive or negative results on the RFT based on the criterion of an angle of deviation greater than 5 deg to indicate visual dependency [8].

## Results

### Self-reported outcome measures

Significant differences were observed on the measures of balance confidence (Activities of Balance Confidence or ABC scale), intensity of dizziness (Visual-Vertigo Analog Scale or VVAS), impact of dizziness on daily activities (Dizziness Handicap Inventory or DHI), and frequency of symptoms (Vertigo Symptoms Scale or VSS-SF) (Table 1). The + VVM group had significantly lower scores on the ABC than the −VVM group (t(131.85) = 12.07, p < 0.001). Additionally, the −VVM adults exhibited significantly lower (better) scores than the + VVM group on the DHI, the VVAS, and the VSS-SF (t _DHI_ (174.54) = −17.12, p  <  0.001), (t _VVAS_ (166.68) = − 36.52, p  <  0.001), (t _VSS-SF_ (197.44) = − 13.26, p < 0.001, respectively).

RFT testing indicated that 85% of participants in the + VVM group and 80% of participants in the -VVM group tested positive for visual dependence; because this result was not significantly different, the impact of visual dependence was not explored further. It should be noted, however, that the angle of deviation from vertical reported on the RFT was significantly higher for the + VVM group than the − VVM group (t(23.30) = − 3.11, p = 0.004).

## Postural acceleration measures

**Fig. 2:**
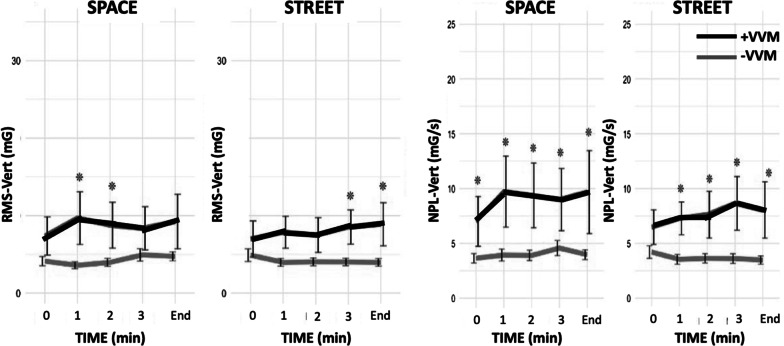
Mean and standard deviations of the RMS (left) and NPL (right) responses in the vertical plane for the + VVM (black line) and − VVM (grey line) participants across the period of the trial during the two visual motion scenes (SPACE and STREET). An asterisk (*) indicates a statistically significant difference between groups (see Table 3 for values). Root mean square (RMS); Normalized path length (NPL); Vertical (VERT); Positive visual-vestibular mismatch (+ VVM); Negative visual-vestibular mismatch (−VVM)

The six postural acceleration measures (i.e., RMS and NPL each in ML, AP, and VERT axes) revealed large variability between the two groups across the three minutes of exposure to the VR environment (Fig. 2). A Wilcoxon signed-rank test revealed significant differences across time between the + VVM and −VVM groups, primarily in the vertical plane of motion. There was also a significant difference with a large effect size at the initiation of STREET motion in the measures of RMS-ML (W = 94, p = 0.01, r = 0.5) and NPL-ML (W = 119, p = 0.01, r = 0.7).

RMS and NPL measures in the AP and ML directions did not exhibit significant differences in either environment (Table 2). There were differing effects of the SPACE and STREET environments on the RMS-VERT measures of the two groups (Fig. 2). A large effect size was observed in the RMS-VERT group means in SPACE (t(40) = − 2.30, p = 0.02, d = 0.78) and in STREET (t(40) = − 2.63, p = 0.01, d = 0.89). Significant differences between groups were also seen in NPL-VERT with medium to large effect sizes across the whole trial period during both SPACE (t(40) = − 2.54, p = 0.01, d = 0.43) and STREET (t(40) = -3.02, p = 0.004, d = 0.87) (Fig. 2 and Table 3).

**Table 2:** Means ± standard deviation (SD) and statistical comparisons of postural measures across the two groups for the whole trial in each of the two virtual scenes

		+ VVM	−VVM		
Measure	Scene	Mean ± SD	Mean ± SD	t-statistic	p-value
RMS AP	Space	12.65 ± 5.96	12.23 ± 6.71	− 0.18	0.85
	Street	12.00 ± 7.85	12.27 ± 7.65	0.14	0.88
RMS ML	Space	10.67 ± 3.56	10.21 ± 5.86	− 0.13	0.89
	Street	11.10 ± 2.67	9.37 ± 7.46	− 0.81	0.42
RMS VERT	Space	**8.93 ± 8.92**	**4.16 ± 2.00**	− **2.30**	0.02*
	Street	**7.96 ± 9.64**	**4.01 ± 6.78**	− **2.63**	0.01*
NPL AP	Space	11.50 ± 4.59	10.14 ± 7.98	− 0.67	0.50
	Street	10.62 ± 5.06	9.72 ± 10.28	− 0.60	0.54
NPL ML	Space	10.34 ± 3.42	9.19 ± 4.85	− 0.39	0.69
	Street	10.49 ± 5.64	7.49 ± 5.40	− 1.71	0.09
NPL VERT	Space	**9.36 ± 8.20**	**4.13 ± 2.73**	− **2.54**	0.01*
	Street	**7.85 ± 8.91**	**3.60 ± 4.47**	− **3.02**	0.004*

*Significant differences are in bold

Positive visual-vestibular mismatch (+ VVM); Negative visual-vestibular mismatch (− VVM); Root mean square (RMS); Normalized path length (NPL); Anteroposterior (AP); Mediolateral (ML); Vertical (VERT)

**Table 3:** Confidence interval and effect size of NPL-VERT with respect to time in the two visual environments

Scene	Statistic	0 min	1 min	2 min	3 min	End
SPACE	*p*-value	0.02^*^	0.01^**^	0.01^**^	0.03^*^	0.01^**^
	CI 95%	− 2.98, − 0.21	− 6.44, − 0.67	− 5.45, − 0.42	− 4.90, − 0.17	− 4.12, − 0.68
	Effect size	0.47 (Medium)	0.58 (Large)	0.51 (Large)	0.42 (Medium)	0.53 (Medium)
STREET	*p*-value	0.12	0.01^**^	0.02^*^	0.01^**^	0.01^**^
	CI 95%	− 3.47, 0.36	− 5.52, − 0.68	− 4.85, − 0.31	− 5.54, − 0.89	− 4.41, − 0.72
	Effect size	0.43 (Medium)	0.57 (Large)	0.46 (Large)	0.56 (Large)	0.54 (Large)

*p < 0.05 ** p < 0.01 *** p < 0.001

### EDA measures

**Fig. 3:**
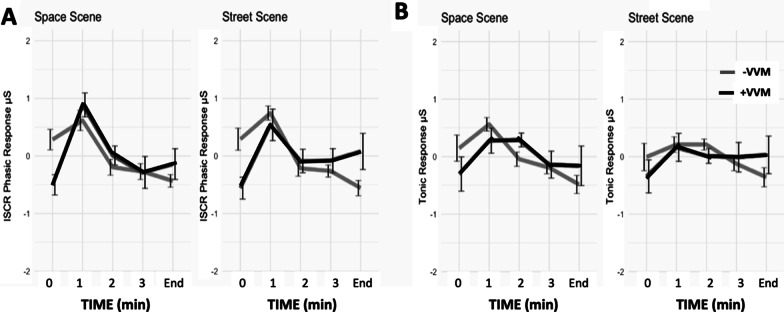
**A** Phasic (EDA ISCR responses of the + VVM positive (black line) and −VVM (grey line) groups across the trial period of the virtual SPACE and STREET environments. **B** Tonic EDA responses of the + VVM positive (black line) and −VVM (grey line) groups across trial period of the virtual SPACE and STREET environments. Positive visual-vestibular mismatch (+ VVM); Negative visual-vestibular mismatch (−VVM)

The −VVM group presented with higher tonic levels of EDA than the + VVM group (Fig. 3). A significant fixed effect of time was observed (F(4,417) = 4.57, p = 0.001) where the + VVM group exhibited an estimated − 0.48 μS less EDA tonic activity than the − VVM group (t(417) = − 4.31, p < 0.001). With both virtual environments, tonic EDA responses of the − VVM group were highest at the initiation of a trial and then dropped below zero by the end of a trial.

A significant fixed effect of time was also observed for the phasic EDA response (F(4,417) = 6.47, p < 0.001). The phasic EDA response in the + VVM group was approximately 0.82 μS greater than the −VVM group (t(417) = 4.35, p < 0.001). The + VVM group started with a lower baseline phasic EDA level than the −VVM group (Fig. 3) in both virtual environments, but this difference was only significant for the SPACE scene (W = 91, p = 0.03, d = 1.05). In the first minute of exposure to virtual scene motion, the phasic EDA response of the + VVM group rose to that of the −VVM group. At the end of the trial, the −VVM group phasic EDA activity dropped to close to the level of the + VVM group (see Fig. 3).

### Effect of visual context

The relationship between the visual environment and the dependent variables (RMS and NPL in the ML, AP, and VERT axes) was explored with LME models across groups (+ VVM and −VVM) and time (Table 4). The STREET environment had a significant effect (F(1360.30) = 19.72, p < 0.001) on trunk motion in the vertical plane (i.e., NPL-VERT). There was an estimated 4.29 μS increase in the + VVM group compared to the −VVM group (t(42) = 2.45, p = 0.01). In the other two planes, NPL values were lower in the + VVM than in −VVM group. The estimated fixed effect revealed that NPL-AP with the STREET scene was approximately − 0.57 μS less in the + VVM than the −VVM group (t(364) = − 2.29, p = 0.02); NPL-ML was − 1.09 μS less in the + VVM than − VVM group (t(368) = − 2.69, p = 0.005).

**Table 4:** Linear mixed model results of time, + VVM, and virtual environment

Terms	Factors	Sum Sq	Mean Sq	Num df	Den df	F value
ISCR Phasic Response	+ VVM	0.02	0.02	1	417	0.03
	Time	45.79	11.44	4	417	18.03***
	+ VVM*Time	16.43	4.10	4	417	6.47***
Tonic Response	+ VVM	0.01	0.01	1	417	0.008
	Time	13.77	3.44	4	417	4.57**
	+ VVM*Time	5.66	1.41	4	417	1.88
NPL AP	+ VVM	0.01	0.01	1	37.21	0.001
	Time	71.27	17.81	4	365.46	2.69*
	STREET	34.67	34.67	1	364.92	5.25*
	+ VVM*Time	56.32	14.08	4	365.46	2.13
NPL ML	+ VVM	7.63	7.63	1	40.16	0.46
	Time	235.72	58.93	4	370.05	3.60**
	STREET	127.20	127.20	1	368.69	7.79**
	+ VVM*Time	78.94	19.73	4	370.05	1.20
NPL VERT	+ VVM	15.73	15.73	1	41.20	4.76*
	Time	43.56	10.89	4	369.70	3.29*
	STREET	45.28	45.28	1	369.35	13.70***
	+ VVM*Time	32.26	8.06	4	369.70	2.44*
RMS AP	+ VVM	3.24	3.23	1	23.21	0.16
	Time	103.04	25.76	4	359.00	1.34
	STREET	9.92	9.92	1	358.09	0.51
	+ VVM*Time	112.07	28.01	4	359.00	1.46
RMS ML	+ VVM	4.43	4.42	1	41.84	0.15
	Time	411.06	102.76	4	372.99	3.49**
	+ VVM*Time	103.97	25.99	4	372.99	0.88
RMS VERT	+ VVM	11.99	11.99	1	40.73	2.93
	STREET	18.30	18.30	1	375.78	4.47*
	+ VVM*STREET	6.13	6.13	1	375.78	1.50

Positive visual-vestibular mismatch (+ VVM); time of exposure to scene motion (Time); Integrated skin conductance response (ISCR); root mean square (RMS); normalized path length (NPL); anteroposterior (AP); mediolateral (ML); vertical (VERT). *p < 0.05 **p < 0.01 ***p < 0.001

**Fig. 4:**
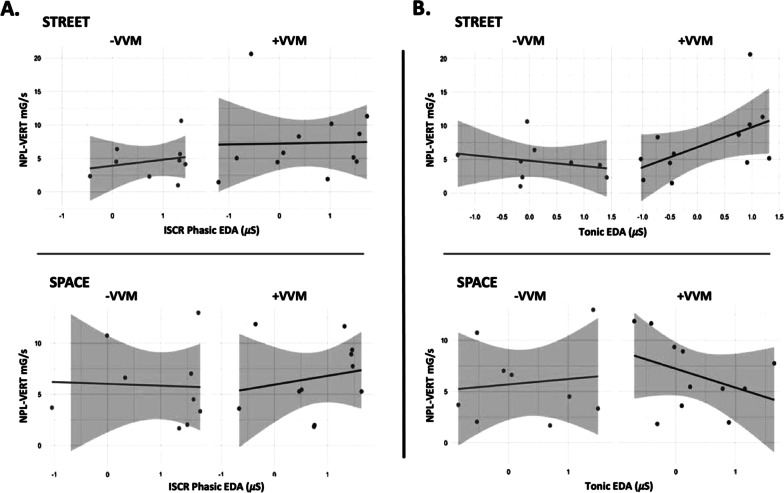
**A** ISCR phasic and **B** tonic electrodermal activity (EDA) (*x-axis*) plotted against normalized path length (NPL) of trunk acceleration in the vertical direction (*y-axis*) while viewing the STREET (top 2 graphs) or SPACE (bottom 2 graphs) virtual environment. Scatterplot on the left in each graph portrays responses of the −VVM group; scatterplot on the right portrays responses of the + VVM group. Positive visual-vestibular mismatch (+ VVM); negative visual-vestibular mismatch (−VVM)

The relationship between visual environment, phasic ISCR, and tonic EDA reveals a possible relationship between visual context, postural, and ANS responses in the + VVM group that is not as evident in the − VVM group (Fig. 4). With + VVM, there is an associated increase in tonic EDA responses and NPL-VERT responses in the STREET scene; ISCR phasic responses of the + VVM group also suggest this association in the SPACE scene. In contrast, there is a distinct *decrease* in NPL-VERT responses as tonic EDA responses *increase* in the SPACE scene.

## Discussion

We have found that EDA and postural sway acceleration responses within a VR environment could distinguish between adults with vestibular migraine and healthy adults [26]. This current study explored whether visual context might be used to further discriminate between adults with vestibular migraine with or without VVM. We hypothesized that the STREET environment would trigger stronger symptoms, and thus, larger EDA and postural responses, than the SPACE environment because it presented a recognizable visual scene with identifiable cues to the performer’s orientation in space [21].

### Impact of visual context

Individuals that tested positive for VVM responded with more frequent postural accelerations in the vertical plane than those testing negative for VVM. In the STREET environment, these frequent vertical plane adjustments were positively associated with a larger tonic EDA. In the SPACE environment, however, postural adjustments decreased as the tonic EDA increased. An association between increased tonic EDA and improved postural performance has been previously reported [24, 25, 48, 49]. Individuals with high levels of tonic EDA were shown to exhibit improved balance confidence and reduced center of pressure displacement in response to sudden external perturbations. High tonic EDA was also positively correlated with higher scores on the ABC scale that are indicative of more postural stability and less severe dizziness [26].

These findings imply that the presence of recognizable objects and verticality cues in the STREET environment supported the attainment of postural control and spatial orientation in individuals with VVM. Conversely, the nebulous visual context and absence of cues to verticality in the SPACE environment was more challenging to the resolution of visual-vestibular conflict.

### Autonomic and vestibular system interrelations

Our results also align with previous reports of visual sensitivity in individuals with vestibular migraine [8]. Although all participants exhibited visual dependency, it is of interest that those in the + VVM group produced the largest deviations from vertical orientation on the RFT. The RFT is a validated tool for otolith-utricular assessment as it measures the degree to which a subject uses available visual cues to locate gravitational vertical [50, 51]. These findings suggest the possibility of canal-otolith dysfunction in + VVM adults. The positive association emerging between vertical acceleration and visual dependence could imply a compensation for this canal-otolith dysfunction by increasing vertical segmental adjustments in order to achieve a perception of verticality.

There is prior evidence that canal-otolith function is strongly linked to both anticipatory and compensatory postural control [52, 53] as is the level of activation in the ANS. Tonic EDA reflects the level of central excitation (i.e., central set) that provides a readiness for expected disturbances. Phasic EDA is the response to a specific event. Therefore, the lower tonic EDA levels exhibited by the + VVM group at the initiation of each trial implies decreased central excitation. Such decreased central excitation has been observed previously in individuals with canal-otolith dysfunction [54]. The highest level of phasic ISCR occurred in both groups when the VR environment was initially projected. This would suggest that either group was capable of matching their response to an anticipated event with what actually did occur in the environment. From these results we might deduce the role of the ANS in postural control as that of resolving symptoms of visual motion sensitivity during exposure to complex visual environments.

### Clinical implications

Both visual context and complexity of the spatial environment surfaces from these findings as important task variables to control with individuals suspected of having VVM. Previous evidence has shown that the amount of uncertainty in visual stimuli strongly influences the amount of induced postural instability [21]. Motion of the visual world was less complex in the SPACE environment as it was presented only in the yaw plane; however, the absence of visual cues to vertical presented a challenge to individuals with VVM. The STREET environment contained multiplanar motion; however, it provided recognizable contexts of a street with 3D objects at randomly generated heights, moving cars, and walking pedestrians. The STREET environment also projected a flow of pedestrians appearing to move toward, away, and next to the participants. Frequent postural adjustments were observed consistent with prior findings from immersive environments containing moving avatars that elicited distinct postural sway behaviors in people with vestibular disorders [55].

There were some limitations of this study. First, cervical (cVEMP) and ocular (oVEMP) vestibular evoked myogenic potential assessments were not available which limited our ability to confirm the integrity of otolith function. Because of COVID-19 restrictions, this study had a small sample size and only one recruitment site which could limit the generalizability of our findings. Lastly, only static balance control was assessed. Future studies that integrate assessment of dynamic balance tasks and measures may provide further insights into the impact of VVM in adults with vestibular disorders.

**Fig. 5:**
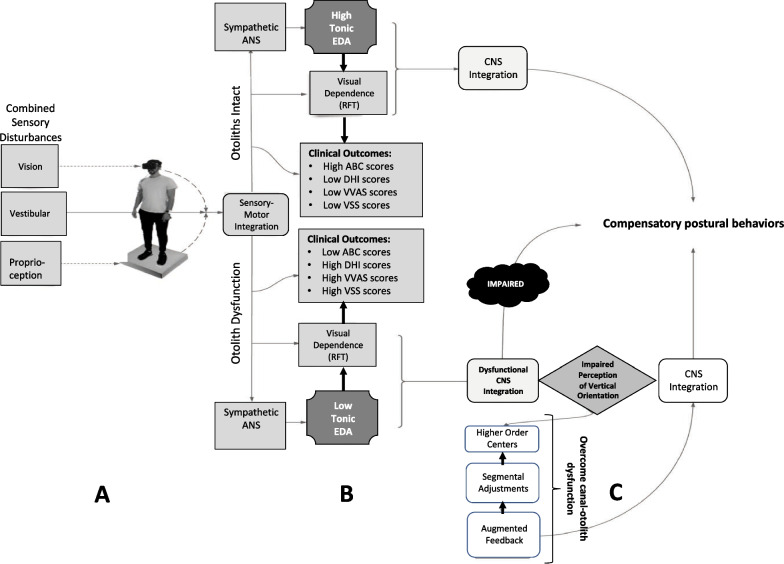
Conceptual schematic summarizing the results of this study and future recommendations for treatment of VVM. **A** Visual, vestibular, and proprioceptive pathways were simultaneously disturbed during the experimental protocol, thereby modifying the sensory-motor integration task. **B** Results of both objective (EDA) and subjective (RFT and outcomes) measures revealed distinct differences between the + VVM and −VVM groups, possibly indicative of dysfunction of the vestibular otoliths with + VVM. **C** Impaired sensory processing in the CNS produces an impaired perception of vertical in the + VVM group, resulting in impaired compensatory postural behaviors. Potential interventions should focus on delivering augmented feedback to both segmental and higher order mechanisms in order to compensate for canal-otolith dysfunction. *VVM*  visual-vestibular mismatch, *CNS*  central nervous system, *ANS*  autonomic nervous system, *EDA*  electrodermal activity, *RFT*  Rod and Frame test, *ABC*  Activities of Balance Confidence scale, *DHI*  Dizziness Handicap Inventory, *VVAS*  Visual Vertigo Analog Scale, *VSS*  Vertigo Symptoms Scale

Nevertheless, the results of this study advance our understanding of the behavioral impact of dizziness with VVM and can help to shape future guidelines for customizing visual environment demands in vestibular rehabilitation. We have developed a conceptual model (Fig. 5) that encapsulates the main results of this study and suggests directions for future intervention. This schematic accentuates the importance of both the vestibular (canal-otolith) system and the autonomic nervous system to compensatory postural control.

As illustrated in Fig. 5, scores from subjective outcome measures combined with measures of tonic EDA activity can provide a meaningful indication of otolith function. The integrity of the otolith organs influences CNS integration processes and, therefore, the ability to produce successful compensatory postural responses. Dysfunction in the otolith organs results in an inaccurate perception of vertical orientation. Segmental adjustments (measured through trunk accelerations) were influenced by perception of the visual environment and might be used as augmented feedback to enhance somatosensory information. This would overwhelm canal-otolith disinformation and bias the performer toward successful postural behaviors.

## Conclusions

The results of this study support our hypothesis that vestibular  and autonomic systems are jointly responsible for postural control and spatial orientation in complex visual environments. When combined with parameterized visual environments, quantitative measures of autonomic nervous system responses can support diagnoses of VVM. Scores from subjective clinical outcome measures combined with measures of tonic EDA activity can provide a meaningful indication of otolith function. The positive association between vertical acceleration, tonic EDA, and visual dependence suggests that vertical postural adjustments may be used to compensate for dysfunction in the vestibular labyrinths, however, clinicians need to consider the context of the visual environment when testing or treating VVM.

## Data Availability

The datasets generated and analysed during the current study are available from the corresponding author on reasonable request.

## Abbreviations

ABC: Activities of balance confidence
ANS: Autonomic nervous system
AP: Antero-posterior plane
CDA: Continuous decomposition analysis
CNS: Central nervous system
cVEMP: Cervical vestibular evoked myogenic potential
DHI: Dizziness handicap inventory
EDA: Electrodermal activity
FOV: Field of view
GSR: Galvanic skin response
HMD: Head mounted display
IMU: Inertial measurement unit
ISCR: Integrated skin conductance response
LME: Linear mixed effect
ML: Medio-lateral plane
NPL: Normalized path length
oVEMP: Ocular vestibular evoked myogenic potential
RFT: Rod and frame test
RMS: Root mean square
S: Seconds
SCL: Skin conductance level
SCR: Skin conductance response
SPACE: Virtual projection of a space scene
STREET: Virtual projection of a pedestrian crossing scene
VERT: Vertical plane
VR: Virtual reality
VSS-SF: Vertigo symptom scale-short form
VVAS: Visual vertigo analog scale
VVM: Visual-vestibular mismatch
−VVM: Individuals with vestibular migraine without VVM
+ VVM: Individuals with vestibular migraine with VVM
VVMQ: Visual-vestibular mismatch questionnaire
$$\mathrm{\mu S}$$: Microsiemens
